# Wave propagation through disordered media without backscattering and intensity variations

**DOI:** 10.1038/lsa.2017.35

**Published:** 2017-09-08

**Authors:** Konstantinos G Makris, Andre Brandstötter, Philipp Ambichl, Ziad H Musslimani, Stefan Rotter

**Affiliations:** 1 Crete Center for Quantum Complexity and Nanotechnology, Department of Physics, University of Crete, Heraklion 71003, Greece; 2 Institute for Theoretical Physics, Vienna University of Technology (TU-Wien), Vienna 1040, Austria; 3 Department of Mathematics, Florida State University, Tallahassee, FL 32306, USA

**Keywords:** Anderson localisation, complex media, non-Hermitian physics, scattering

## Abstract

A fundamental manifestation of wave scattering in a disordered medium is the highly complex intensity pattern the waves acquire due to multi-path interference. Here we show that these intensity variations can be entirely suppressed by adding disorder-specific gain and loss components to the medium. The resulting constant-intensity waves in such non-Hermitian scattering landscapes are free of any backscattering and feature perfect transmission through the disorder. An experimental demonstration of these unique wave states is envisioned based on spatially modulated pump beams that can flexibly control the gain and loss components in an active medium.

## Introduction

The scattering of waves through disordered media is a paradigmatic phenomenon that has captured the interest of various communities for quite some time now^1, 2, 3^. Among the many important physical aspects of wave propagation that have been studied the phenomenon of Anderson localisation has received particular attention^4, 5, 6, 7, 8, 9, 10, 11, 12^. While much work has been invested into understanding the ‘statistical’ properties of the corresponding wave phenomena^13^ there has recently been a surge of interest in controlling the scattering of waves through ‘individual’ systems for specific purposes such as detection, imaging, and efficient transmission across disordered materials^14, 15^. Remarkable progress in these endeavours has recently been made in the optical domain, largely due to the availability of spatial light modulators and new concepts for how to apply them on turbid media^16, 17^. In a first generation of corresponding experiments the focus was laid on shaping the input wave front impinging on an immutable disordered sample such as to achieve a desired output, like a spatial or temporal focus behind the medium^18, 19, 20, 21^. More recent studies focused instead on controlling the medium itself, for example, through the material fabrication process^22^ or through a spatially modulated pumping^23^, leading, for example, to a versatile control of random and micro-cavity lasers^24, 25, 26, 27, 28^.

Largely in parallel to these efforts on disordered media, it was recently realised that materials and devices can get entirely new functionalities when adding to them a suitably arranged combination of gain and loss. In particular, structures with a so-called parity time (PT) symmetry^29, 30^, were recently introduced theoretically^31, 32, 33^ and experimentally^34, 35^ in the context of paraxial waveguide optics. On the basis of a delicate balance between gain and loss, these synthetic structures exhibit rich and unconventional behaviour, holding promise for numerous applications in nano photonics and lasers^36^. In particular, the relation between coherent perfect absorption and scattering through PT-cavities^37^ as well as unidirectional invisibility in PT-gratings^38^ have attracted a lot of attention. Symmetry breaking in fibre loop optical networks^39^, PT-scattering structures^40^ and periodic PT-systems as new types of metamaterials^41^ are also active research directions in this new field on PT-optics. Along with these activities, another direction is that of complex lasers that rely on the concepts of PT symmetry breaking and exceptional points. Such synthetic lasers with novel characteristics, are based on loss engineering^42^. More specifically, coupled PT-symmetric micro disk lasers can lead to optical diodes^43^, single-mode microring lasers^44^, synthetic PT-lasers^45^, loss-induced lasing^46, 47^ and lasers with chiral modes^48, 49^. More recent developments include large scale exceptional points in multilayer optical geometries^50^, transient growth in non-normal lossy potentials^51^, modulation instabilities in non-Hermitian structures^52^, non-Hermitian phase matching in optical parametric oscillators^53^, higher-order exceptional points^54^, protocols for asymmetric mode switching based on encircling exceptional points dynamically^55, 56^, and directional cloaking based on non-Hermitian potentials^57^.

Here we will build on the advances that were made in both of the above research fields with the aim to combine them in a novel and potentially very useful way. We show that for a general disordered medium, given by a distribution of the real part of the refractive index *n*_R_(*x*), a corresponding distribution of its imaginary part *n*_I_(*x*) can be found, such that a wave propagating through this medium will feature a constant intensity throughout the entire non-uniform scattering landscape. In other words, we demonstrate that adding a judiciously chosen distribution of gain and loss to a disordered medium will make waves lose all of their internal intensity variations such that they propagate through the disorder without any back-reflection.

## Materials and methods

### Scattering states without internal intensity variations

The solution strategy that we explore for this purpose is based on the one-dimensional Helmholtz Equation that describes time-independent scattering of a linearly polarised electric field *ψ*(*x*) both in forward and in backward direction,


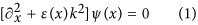


Here *ε*(*x*) is the dielectric permittivity function varying along the spatial coordinate *x*, *k*=2*π*/*λ* is the wavenumber (with *λ* being the free space wavelength) and ∂_*x*_≡d/d*x*. The dielectric function is complex thus *ε*(*x*)=[*n*_R_(*x*)+*in*_I_(*x*)]^2^. In general, when a plane wave is incident on a spatially varying distribution *ε*(*x*), interference takes place between the waves propagating forward and backward. As a result, a complex interference pattern is produced with spatial variations on its intensity. As we will now show, this fundamental physical picture can be quite different in the case of non-Hermitian cavities with loss and/or gain.

To jump right to the heart of the matter, we start with an ansatz for a constant-intensity (CI) wave with unit amplitude, *ψ*(*x*)=exp[*iS*(*x*)], where *S*(*x*) is a real valued function. Because of the obvious relation to the semiclassical approximation by Wentzel-Kramers-Brillouin (WKB)^58^, we will derive the CI-solution of the Helmholtz Equation (1) in the bulk, by demanding that the ansatz *ψ*(*x*)=exp[*iS*(*x*)] has to be exact in the first order WKB-approximation. As a result of this analysis (see Supplementary Information for details) we obtain the following non-Hermitian dielectric function,


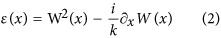


that supports a corresponding CI-solution 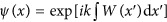 at wavenumber *k*, which solves Equation (1) exactly and is valid for the whole bulk space. At this point we have to emphasise, that the above exact WKB analysis is generally valid (not only in the geometric optics limit). The fact that *W*(*x*) can be chosen arbitrarily, with no limitations on its spatial complexity (apart from smoothness), is a key asset of this approach, making it very generally applicable. From this result it is also clear that for vanishing imaginary part [*W*(*x*)=const.], the dielectric function as defined in Equation (2), reduces to *ε*(*x*)=const., resulting in the familiar plane wave in free space. We emphasise that our approach works not only when the functional profile *W*(*x*) is known from the outset. Also when a refractive index distribution *n*_R_(*x*) is given, the corresponding gain-loss profile *n*_I_(*x*) can be determined (see Supplementary Information for details).

Furthermore, it can be shown that CI-waves can also be found for all dielectric functions that are described by Equation (2) in a finite domain *x*∈[−*D*, *D*], bordering on free space for *x*<−*D* and *x*>*D*. In this case, the scalar Helmholtz-type Equation (1) admits the following exact CI-scattering state:


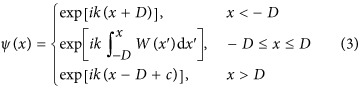


where the integration constant *c* makes sure that the field continuity relations^59^ are satisfied and is given by 
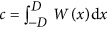
. We note at this point that the wavenumber *k* appearing in Equation (2) is the same as the wavenumber *k* in the above CI-wave solution. In other words, for any value of *k* for which a CI-scattering state is desired, the dielectric function *ε*(*x*) has to be engineered correspondingly. Once *ε*(*x*) is fixed and plane waves with varying values of *k* are impinging on this dielectric structure, a perfectly transmitting CI-solution in general only occurs at the predetermined *k* value inherent in the design of *ε*(*x*). Due to this restriction to a single frequency also no issues arise with the Kramers-Kronig relations.

Most importantly, the solution in Equation (3) does not only feature a constant intensity |*ψ*(*x*)|^2^=1 in the asymptotic regions *x*<–*D* and *x*>*D*, where *ε*(*x*)=1 and simple plane wave propagation is realised, but also inside the finite region of length 2*D* in which the dielectric function varies and the phase-evolution is non-trivial. Regarding the appropriate boundary conditions at *x*=±*D*, it can be shown that the following perfect transmission boundary conditions (zero reflection)^40^,


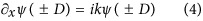


imply the following conditions for the generating function,


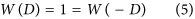


We also emphasise that a CI-wave is associated with a specific incidence direction (incidence is assumed here from the left, *x*<−*D*, in positive *x*-direction). When injecting a plane wave with the same predetermined wavenumber *k* from the other incidence direction (that is, from the right, *x*>*D*, in negative *x*-direction) one still gets perfect transmission simply due to Lorentz-reciprocity. In addition, however, one will also get finite reflection and non-trivial intensity variations inside the potential (|*x*|≤*D*).

### Non-Hermitian scattering methods

To elucidate the above ideas, we consider in the section ‘Results and Discussions’ several specific examples of index distributions and study the CI-waves they give rise to. For these calculations, a transfer matrix method^59^ was used for TE-linearly polarised optical waves along with an effective Hamiltonian approach^40^. More specifically, the transfer matrix method is valid for piecewise refractive index distributions. To apply such an approach to our scattering problem, we discretised the continuous potential into many slabs of almost constant refractive index, and then applied the transfer matrix method. Another technique we used was that of the effective Hamiltonian. The wave Equation (1), 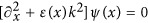, can be written as a generalised eigenvalue problem for the potential of Equation (2) for a given *W*(*x*). More specifically we have: 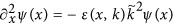. This generalised eigenvalue problem is non-Hermitian due to the perfect transmission boundary conditions. Notice that only one of its eigenmodes will be the CI-state and satisfy the relation 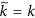. We have compared the two different approaches for specific optical structures and they give identical results.

## Results and discussion

**Figure 1:**
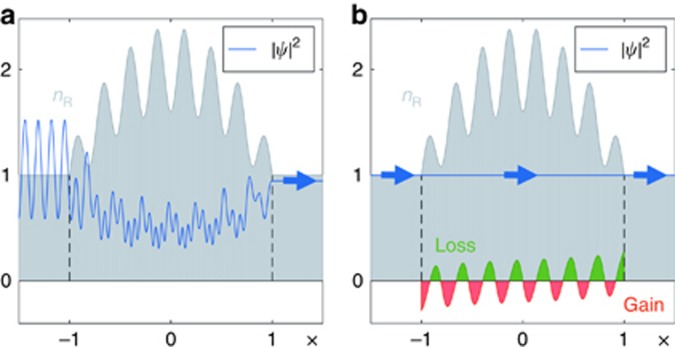
(**a**) Scattering wave function intensity (blue line) in a Hermitian refractive index distribution for an incident plane wave (from the left) with a specific normalised wavenumber *k*=2*π*/0.26=24.15. (**b**) Intensity of the CI-wave for the corresponding non-Hermitian refractive index *n*(*x*) and the same incident plane wave. The real part of the refractive index is shown in grey, whereas its imaginary part is coloured in green (loss) and red (gain). For illustration purposes the imaginary part in **b** was multiplied by a factor of 2. The calculations were performed using the transfer matrix approach.

As a first example, we assume *W*(*x*) to be a parabolic function modulated with a cosine, namely *W*(*x*)=[1−0.2 cos(15*πx*/2)](2−*x*^2^). The corresponding real part of the refractive index distribution *n*_R_(*x*) is shown as the grey shaded area in Figure 1. A wave impinging on this dielectric structure composed of only *n*_R_(*x*) is partly reflected and features a highly oscillatory profile, see Figure 1a. Quite in contrast, when adding also the gain and loss inherent in the imaginary index component *n*_I_(*x*) derived from *W*(*x*) (see green and red regions in Figure 1b), the resulting scattering state is fully transmitted and features a constant intensity. Because of the boundary conditions, *W*(*x*) must be symmetric at the end points of the cavity, resulting in an anti-symmetric distribution of *n*_I_(*x*). Our example shows that for a plane wave at an arbitrary incident wavenumber *k*, we can find the corresponding gain-loss landscape (from Equation (2)), such that this wave will fully penetrate the scattering medium without forming any spatial variations in its intensity pattern.

### Perfect transmission through disorder

The most striking application of CI-waves is realised for the case of disordered environments, which is also the focus of our work. We know, for example, that in strongly scattering disordered media Anderson localisation occurs, resulting in an exponential decrease of the transmittance *T*=|*t*|^2^ for structures with sizes greater than the localisation length 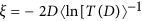. For a given real and disordered index of refraction in the localised regime close to unit transmittance is thus very unlikely and occurs only at well-isolated, sharply resonant wavenumbers that are difficult to achieve experimentally^60, 61^. Our approach now allows to turn this behaviour upside down—not only in the sense that we can engineer unit transmission at any predetermined value of the wavenumber *k* but also that we can create scattering states that have constant intensity in a strongly disordered environment which would usually give rise to the most dramatic intensity fluctuations known in wave physics.

**Figure 2:**
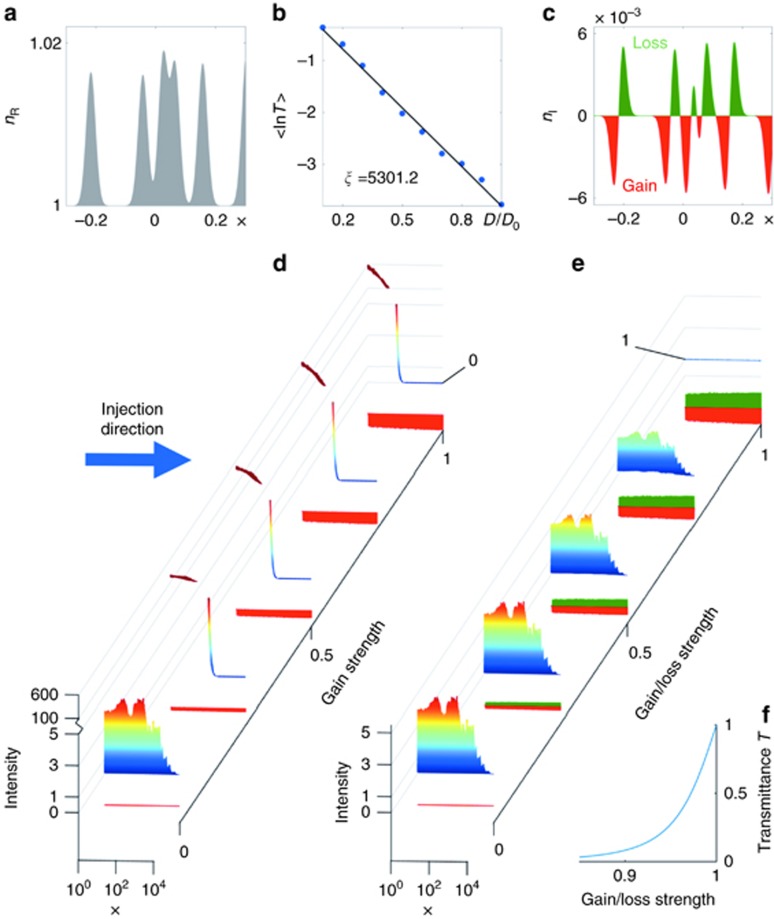
A strongly disordered potential consisting of *N*=99 000 Gaussian scatterers is considered in the region −*D*_0_<*x*<*D*_0_ with *D*_0_=5000. (**a**) The corresponding refractive index distribution *n*_R_(*x*) in a small interval of *x* is shown. (**b**) Exponential suppression of the transmittance *T* with localization length *ξ* in this system for variable length of the disordered region *D*. (**c**) Imaginary part of the refractive index *n*_I_(*x*) following from the CI design principle (*n*_I_(*x*) is matched to the real index distribution in **a**). (**d** and **e**) Scattering wave functions for the disordered region as a function of the gain–loss strength parameter *a*, for the gain-only and gain–loss potential, respectively. In both cases, an incident plane wave with normalised wavenumber *k*=2*π*/0.1=62.8 is considered (from left to right), and the *x* axis is represented in logarithmic scale. The CI-wave can be clearly seen for the full gain–loss strength (*a*=1) in (**e**). (**f**) The transmittance *T* is shown here for different gain–loss strength *a*, indicating a smooth transition to perfect transmission *T*=1 at *a*=1.

We illustrate our results for the disordered one-dimensional slab shown in Figure 2, where a refractive index distribution following Equation (2) is considered with a tunable imaginary component, *ε*(*x*)=[*n*_R_(*x*)+*ian*_I_(*x*)]^2^ (the tunable parameter *a* controls the overall amplitude of gain and loss). From Equation (2), one can understand that CI-scattering-states exist only for *a*=1. In particular, we choose the generating function *W*(*x*) to be a superposition of *N* Gaussian functions of the same width *d*, but centred around random positions *c*_*n*_ and having random amplitudes *r*_*n*_. More specifically, we consider 
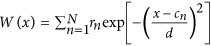
, where *c*_*n*_ and *r*_*n*_ are random variables. This leads us to the following analytical expression of a CI-state *ψ*(*x*) in a disordered medium:


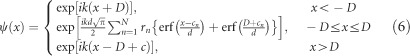


where the error function is defined as follows: 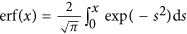, and the constant *c* is defined as in Equation (3). For *a*=0 the refractive index is Hermitian, whereas for *a*=1 CI-waves exist. The refractive index distribution of such a non-Hermitian disordered medium is depicted in Figure 2a, and the localisation length *ξ* of the Hermitian refractive index (*a*=0) is depicted in Figure 2b. The imaginary part (gain and loss) of the refractive index distribution that leads (based on Equation (2)) to a CI-state is depicted in Figure 2c. As is physically expected, without the gain and loss distribution, the system reflects almost all waves due to localisation. Furthermore, adding first only the gain part of the refractive index distribution that leads to a CI-state (see Figure 2c) still results in highly oscillatory scattering wave functions with finite reflectance for all values of the gain-loss amplitude *a* (from 0 to 1), as is illustrated in Figure 2d. Quite counterintuitively, adding also the loss part of the refractive index distribution leads to perfect transmission without any intensity variations for *a*=1, as demonstrated in Figure 2e. By varying the gain-loss amplitude *a*, as in Figure 2e, we can thus see a smooth transition from the Anderson localisation regime (at *a*=0) to perfect transmission with constant intensity (at *a*=1). Figure 2f shows this transition for the transmittance *T*(*a*), indicating the robustness of our approach as well as the fact that already smaller values of *a*<1 lead to a transmittance that is several orders of magnitude larger than in the absence of gain and loss (*T*≈2 × 10^−3^ for *a*=0). We thus find that the presence of gain and loss in a scattering environment can completely suppress the localisation of waves due to multiple scattering, leading to a delocalised state with a constant intensity and perfect transmission.

A crucial question is the physical values of gain and loss required to observe and realise the proposed CI-waves. It turns out that these values depend directly on the slope and amplitude of the refractive index distribution, as well as on the wavelength of operation, as we can see from Equation (2). The maximum value of gain/loss for the system shown in Figure 2 is at max(n_*I*_) ≈ 6.7 × 10^−3^ with the length of the disorder region being 6 cm. With a wavelength of *λ*=0.58 μm this would correspond (for a variation of Δ*n*_R_ ≈ 2 × 10^−2^) to a gain value of ~1450 cm^−1^. Typical example gain media at this wavelength that are also implementable in a practical experimental setting are, for example, Rhodamine (6 G) dye materials, commonly used in active plasmonics^62^ and in opto-fluidic random lasers^24^. The above gain values are, however, quite high for organic media such that in an experimental implementation smoother refractive index distributions, *n*_R_(*x*), are more realistic, for which the corresponding gain values are smaller. Since, in turn, the localisation length *ξ* will then be larger (in the absence of gain and loss), also longer disorder regions will be required to see the transition from localised states to CI-waves. We emphasize, however, that our approach is completely scalable also to other wavelengths and gain media. First practical applications that we anticipate will probably also be focused on exploring only a subpart of the above crossover.

### Discrete disordered systems

**Figure 3:**
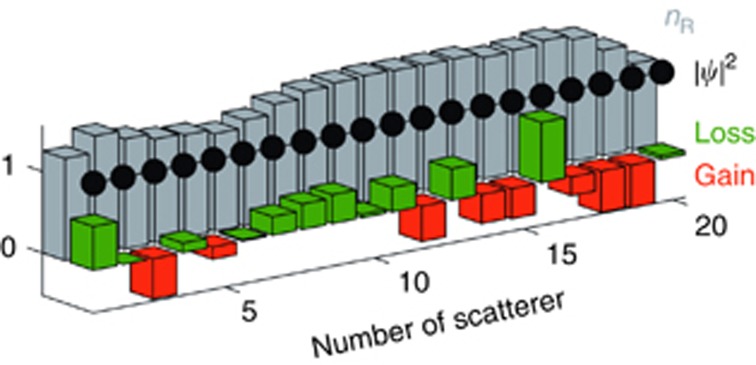
Disordered chain of discrete scatterers with an incoming plane wave from the left. The real part (grey) as well as the gain (red) and loss (green) components of the refractive index are shown for each scatterer. The corresponding discrete CI-wave is depicted with black dots. The normalised parameters used are *M*=20, *ω*=12, *L*=2 and Δ*x*=*L*/(*M*−1).

Certainly the most challenging aspect of CI-waves in terms of their experimental realisation is the fabrication of a specific index distribution with gain and loss^63^. To overcome such inherent difficulties, we study here also the existence of CI-waves in a system of discrete scatterers, like the one presented in Figure 3. Such a setup is composed of many discrete elements (cavities) with gain or loss and a specific real refractive index distribution. The analytic solution of Equation (2) is still valid in the discrete version of the Helmholtz-type wave Equation:


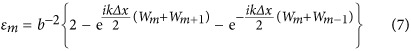


and


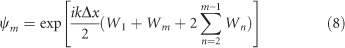


where *ε*_*m*_ is the permittivity of the *m*th scatterer, *b*=*ω*Δ*x*, *ω*^2^=2[1−cos(*k*Δ*x*)]/Δ*x*^2^ and *m*=1,…, *M*. In addition, the perfect transmission boundary conditions imposed at the end points of the discrete chain of scatterers *ψ*_0_=*ψ*_1_ exp(−*ik*Δ*x*), and *ψ*_*M*+1_=*ψ*_*M*_ exp(*ik*Δ*x*) as well as the relation *ω*Δ*x*<2 must always hold. We consider a specific example in Figure 3 of *M* elements that form a one-dimensional disordered chain. By adding gain or loss onto the sites as prescribed by Equation (7) an incoming wave from the left will have the same constant intensity on all of these sites.

### PT symmetry and mean reality condition

So far we have not discussed the relation of the non-Hermitian distribution (Equation (2)) that supports CI-waves with PT symmetry. For the special case that the generating function is even with respect to *x*, namely *W*(*x*)=*W*(−*x*), the dielectric function turns out to be PT-symmetric since *ε*(*x*)=ε*(−*x*). In other words, our approach is rather general and the only restrictions are the permittivity distribution (Equation (2)) and the boundary conditions (Equation (5)) for *W*. Keep in mind that Re[*ε*(*x*)] could in principle also be negative—at least there is no restriction from the mathematical point of view. Since we wish to study relevant physical materials that are easily accessible also experimentally, we choose our *W*(*x*) such that we have Re[*ε*(*x*)]>1 and also *n*_R_(*x*)>1. A direct consequence of these two restrictions is that the spatial average gain-loss over the scattering region is zero, 
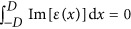
. In terms of the refractive index, the evaluation of the integral 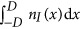 depends on the symmetry of *W*(*x*). For example, if *W*(*x*) is an even function of *x*, then the refractive index is PT-symmetric, and 
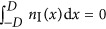
. It would be interesting to explore if this condition of ‘mean reality’ also has other interesting consequences that go beyond the restrictions imposed by PT symmetry.

### Effect of wavenumber detuning and gain saturation

**Figure 4:**
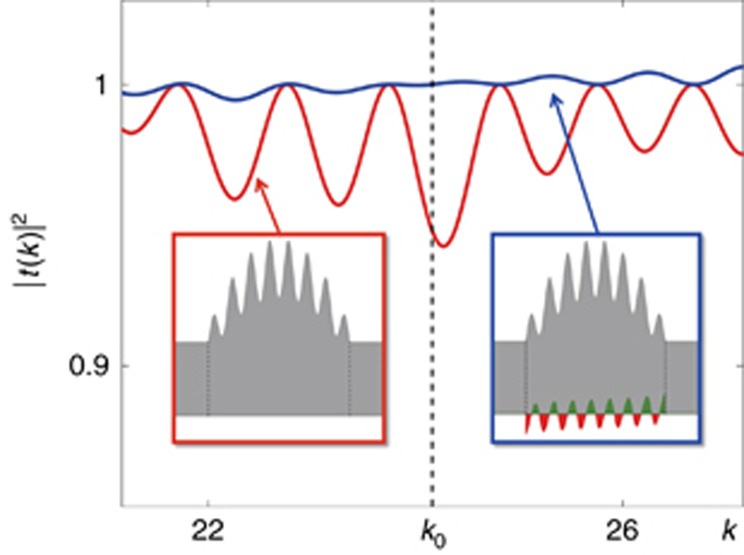
Effect of incident wavenumber detuning in a narrow wavelength window, on the transmittance through the potential of Figure 1b (blue line). The Hermitian case is plotted for comparison (red line). The two insets illustrate the complex refractive index distributions.

As we have already shown analytically and numerically, for a fixed refractive index determined by Equation (2), a CI-wave at the corresponding wavenumber *k*_0_ exists. A natural question one may ask at this point is what happens to incident plane waves with detuned wavenumbers *k*_0_±Δ*k*_0_ (Figure 4; when considering active materials that are characterised by approximately flat dispersion curves near the values of the wavelength of operation). Naively, one may expect that the emergence of CI-waves is a sharp resonance phenomenon, so that waves with a slight detuning in the wavenumber *k*_0_ should show a completely different behaviour as, for example, around a resonance in a Fabry-Pérot interferometer^59^. This picture turns out to be misleading on several levels. Since the CI-wave function at position *x*, 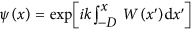, only depends on the generating function *W*(*x*') evaluated at values *x*'<*x*, one can easily truncate the system at any point *x* and still get a CI-wave provided one continues the system for all *x*>*x*' with a constant generating function that has the same value as at the point of truncation. This behaviour indicates that a refractive index profile that supports CI-waves is not only reflectionless in total but also unidirectional at any point inside a given structure. Perfect transmission in such systems is thus not a resonance phenomenon (as resulting from a back and forth propagation of waves), suggesting that CI-waves are stable against changes of the incident wavelength. To check this explicitly, we numerically calculated the average resonance width of the transmission spectrum |*t*(*k*)| of the Hermitian system in Figure 1, 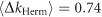, in an interval 
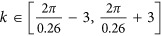
, with minimum transmission |*t*(*k*)_min_|=0.94, as is shown in Figure 4. The transmission of waves through the corresponding non-Hermitian CI-refractive index (that of Figure 1b) stays larger than 0.99 over the entire *k*-interval, confirming our prediction. Another important point to make is that one can easily achieve a transmission equal to one in a non-Hermitian system just by adding enough gain to it. In the scattering landscapes that we consider here, however, the net average amplification is zero, since 
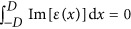
 and the intensity is equally distributed everywhere.

In realistic gain materials, high enough field amplitudes lead inevitably to gain saturation. A natural question to ask is if CI-waves exist also in this nonlinear regime. As we show in the Supplementary Information, one can analytically derive CI-scattering-states by solving the corresponding nonlinear Helmholtz equation. The difference in this case is that the CI amplitude is specific for the parameters of the gain material.

### Connections to the literature

The results presented above also have several interesting connections to earlier insights from the literature. Perhaps the first mention of CI-waves was made in laser physics in an under-appreciated work by Yariv *et al.*^64^, in which it was shown that modes in distributed feedback (DFB) lasers can be engineered to have a constant intensity throughout the entire laser device—a feature that was proposed as a strategy to overcome spatial and spectral lasing instabilities^65, 66^. The design principle for CI-waves in these DFB lasers was, however, restricted to periodic potential structures without any incoming wave and is thus orthogonal to our own approach. In a recent work, CI-waves were presented as solutions of the paraxial wave Equation where the ‘Wadati potential’, previously introduced in Ref. 67, varies only transversely to the propagation direction^52^. The paraxial approximation employed in that work, however, excludes any backscattering from the potential and the incoming wave had to be engineered through wave front shaping to yield the desired CI-solution. The approach presented here has the clear advantage that it does not rely on any approximation, that no wave front shaping of the incoming wave is necessary and that it can be applied to arbitrary, even disordered potentials with an unspecified amount of backscattering. We also mention in this context that during the last few years non-Hermitian potentials without PT symmetry that yield real propagation constants (including similar ones as in our Equation (2)) have been studied (see Refs 68, 69, 70, 71, 72). In our own work we are, however, not concerned with a phase transition to complex eigenvalues, but rather focus on the unique possibility to achieve perfect transmission and a suppression of any intensity variations in disordered media. For this purpose it is clearly essential that, in contrast to these earlier works, we address here the full scattering problem including backscattering. Last but not least we also highlight that our approach opens up a new and promising way to apply the WKB-approximation to potentials such as those with a short-range disorder, that usually fall completely outside the scope of this well-studied approximation.

## Conclusions

In conclusion, we examine the existence and the properties of a novel type of waves, the CI-waves in one-dimensional non-Hermitian optical slab geometries with and without disorder. For any wavenumber *k* of a plane wave incident on a real refractive index distribution, we can identify a corresponding gain-loss profile that allows for CI-waves to propagate through such a scattering landscape without any reflection. Most importantly, we found the gain-loss profiles that need to be added to any disordered system such as to completely overcome the strong backscattering and the intensity variations that usually occur in such media. As a first step towards an experimental realisation we propose to study chains of discrete scatterers with gain and loss that can nowadays be routinely fabricated in the laboratory.
