# Supplementary material for: Wave propagation through disordered media without backscattering and intensity variations
Source: Light Sci Appl. 2017 Sep 8;6(9):e17035–. doi: 10.1038/lsa.2017.35 (PMC6062329; doi:10.1038/lsa.2017.35)
Supplement: Supplementary Information [file lsa201735x1.doc]

Supplemental Information for

"Wave propagation through disordered media without backscattering and intensity variations"

Konstantinos G. Makris^1^, Andre Brandstötter^2^, Philipp Ambichl^2^, Ziad H. Musslimani^3^, and Stefan Rotter^2^

^1^Crete Center for Quantum Complexity and Nanotechnology, Department of Physics, University of Crete, Heraklion 71003, Greece

^2^Institute for Theoretical Physics, Vienna University of Technology, Vienna 1040, Austria

^3^Department of Mathematics, Florida State University, Tallahassee, Florida 32306, USA

Email: KGM, makris@physics.uoc.gr; AB, andre.brandstoetter@tuwien.ac.at;
 PA, philipp.ambichl@tuwien.ac.at; ZHM, muslimani@math.fsu.edu; SR, stefan.rotter@tuwien.ac.at
Correspondence: KGM, Email: makris@physics.uoc.gr, Phone: 30-2810-394227

**WKB analysis.** Expanding the function $S(x)$ in powers of a small parameter $\delta$, $S(x)=\delta^{-1}\sum_{n=0}^{\infty} \delta^{n}S_{n}(x)$, and inserting it into the Helmholtz Eq. (1) to leading order, we can show that in the limit of $\delta\to0$, $\delta$ scales with $k^{-1}$. Setting $\delta=1/k$ and collecting terms with the same power of $k$, we can write down the two dominant terms, namely $k^{2}=1/\delta^{2}$, and $k^{1}=1/\delta^{1}$. To each of these terms a corresponding equation is found:

$\mathrm{Re}[\varepsilon(x)]+i\mathrm{Im}[\varepsilon(x)]-[S'_{0}(x)]^{2}=0$ (0.1)

. (0.2)

The exactness requirement of our ansatz necessitates that all terms $S_{n>0}$ are zero and the demand for constant intensity of $\psi(x)$ calls for a real-valued $S_{0}(x)$. Both conditions can be fulfilled by choosing $\mathrm{Im}[\varepsilon(x)]=-S''_{0}(x)/k$ such that the term $\mathrm{Im}[\varepsilon(x)]$ moves from Eq. (0.1) to Eq. (0.2), leading to $\mathrm{Re}[\varepsilon(x)]=[S'_{0}(x)]^{2}$ and

$S'_{1}(x)=0$. As a result $S_{1}(x)=const.$ and all higher terms are constant as well.

Setting $S'_{0}(x)=W(x)$, we finally obtain the non-Hermitian dielectric function (relative permittivity), as shown in Eq. (2).

**Iteration technique for determination of the gain-loss profile given the $n_{R}(x)$ distribution.** Given the function $W(x)$, the dielectric distribution $\varepsilon(x)$ and the corresponding refractive index distribution can be directly determined and vice versa. The same is, however, not true if, as a starting point, the real part of the refractive index $n_{R}(x)$ is known instead (typical situation in many realistic cases). The reason is that by adding an imaginary part of the refractive index $n_{I}(x)$ (e.g., by pumping), one not only changes $\mathrm{Im}[\varepsilon(x)]$ but also $\mathrm{Re}[\varepsilon(x)]$. In other words, by adding gain and loss to a material, the real part of the dielectric function changes as well. In order to overcome such a problem, we employ an iterative technique, that is based on the following expressions between $W(x)$ and the complex index of refraction: $n^{2}_{R}(x)-n^{2}_{I}(x)=W^{2}(x)$ and $2n_{R}(x)n_{I}(x)=-[\partial_{x}W(x)]/k$. To identify $n_{I}(x)$ and $W(x)$ required for CI-states (that correspond to a specific and given $n_{R}(x)$ distribution), we applied an iterative numerical scheme that allows to determine these unknown distributions, by starting with a reasonable guess function for $W(x)$. In particular, at the $m^{th}$ step of iteration the equations read:

$n_{I}^{(m+1)}=-W^{'(m)}/(2kn_{R})$ (0.3)

$W^{(m+1)}=[n^{2}_{R}-n_{I}^{2(m+1)}]^{1/2}$, (0.4)

where $k$ is the given wavenumber of the incident plane wave. We explicitly checked that the method converges and leads to the desired results.

**Effects of gain saturation.** We prove here that the constant-intensity state can be a scattering eigenstate mode under perfect transmission boundary conditions in a medium with gain saturation^1^. The corresponding nonlinear Helmholtz equation is:

$\frac{d^{2}U}{dx^{2}}+k^{2}[\varepsilon_{R}(x)+i\varepsilon_{I}(x)]U+\frac{ik^{2}P(x)}{1+\Gamma|U|^{2}}U=0$, (0.5)

where $\varepsilon_{I}(x)>0$ describes the material loss in the cavity, $P(x)<0$the pump, and $\Gamma$ the saturation coefficient. We are looking for constant-intensity states of the form

$U(x)=A\mathrm{e}^{ik\int W(x') dx'}$, (0.6)

where the amplitude $A$ of the solution plays an important role now. The nonlinear Eq. (0.5) can be written in the form of Eq. (1) with an effective complex permittivity $\varepsilon_{eff}(x)$, defined as follows:

$\varepsilon_{eff}(x)=\varepsilon_{R}(x)+i[\varepsilon_{I}(x)+\frac{P(x)}{1+\Gamma|U|^{2}}]$. (0.7)

By substitution of Eq. (0.7) into Eq. (0.5) we obtain the following equations:

$\mathrm{Re}[\varepsilon_{eff}(x)]=\varepsilon_{R}(x)=W^{2}(x)$, (0.8)

$\mathrm{Im}[\varepsilon_{eff}(x)]=\varepsilon_{I}(x)+\frac{P(x)}{1+\Gamma A^{2}}=-\frac{1}{k}\frac{dW}{dx}$. (0.9)

Given the auxiliary function $W(x)$ the real part of the permittivity can be easily determined by the above Eq. (0.8). Regarding the imaginary part of the permittivity, and, the pump profile $P(x)$*,* these can be determined as follows:

$\varepsilon_{I}(x)=\left\{\begin{matrix} k^{-1}dW/dx, W'<0, \\ 0, W'>0 \end{matrix} \right.$ (0.10)

$P(x)=\left\{\begin{matrix} (1+\Gamma A^{2})k^{-1}dW/dx W'>0, \\ 0, W'<0. \end{matrix} \right.$ (0.11)

## REFERENCES

1 Premaratne M, Agrawal GP. *Light Propagation in Gain Media: Optical Amplifiers*. Cambridge University Press: Cambridge, 2011.
